# Association of different intensities of physical activity in children with parental support

**DOI:** 10.3389/fpsyg.2025.1600667

**Published:** 2025-07-21

**Authors:** Siyan Wang, Xiuxiu Zhou, Kai Li

**Affiliations:** ^1^Department of Public Sports Education and Research, Guangxi Police College, Nanning, Guangxi, China; ^2^China Jiuquan Satellite Launch Center, Jiuquan, Gansu, China; ^3^College of Sports Industry and Leisure, Nanjing Sport Institute, Nanjing, Jiangsu, China

**Keywords:** parental support, physical activity, different intensities, children, Macau

## Abstract

**Objective:**

To investigate the status of physical activity and parental support of children aged 6–10 years in Macau and to explore the relationship between them.

**Methods:**

The International Physical Activity Questionnaire-Short form (IPAQ-S) and Activity Support Scale for Multiple Groups (ACTS-MG) were used to investigate children’s physical activity time, parental support level, and general demographic information. The height and weight of the children are measured by a portable instrument.

**Results:**

The physical activity of children in Macau is insufficient, does not meet the guidelines of the World Health Organization (WHO). Regarding parents’ support, the average score of physical activity models and community resource utilization is relatively low. Linear regression results showed that the relationship between low physical activity (LPA) and parental support was not statistically significant, and that children with moderate physical activity (MPA) (over all *β* = 0.256, boys *β* = 0.245, girls *β* = 0.279), moderate-to-vigorous physical activity (MVPA) (over all *β* = 0.291, boys *β* = 0.279, girls *β* = 0.312) were significantly positively correlated with logistical support. Boys’ vigorous physical activity (VPA) (*β* = 0.335) was significantly positively correlated with community resource utilization, and girls’ vigorous physical activity (VPA) (*β* = 0.268) was significantly positively correlated with physical activity models.

**Conclusion:**

Macau parents generally pay attention to children’s physical activity endeavors, but the level of children’s physical activity is relatively low. There are certain differences in the relationship between the types of parental support and the physical activity of children of different genders, and the relationship between parental logistical support and children’s physical activities is closer. In future studies, investigators could look at various family interventions and make more targeted plans based on gender and activity intensity.

## 1 Introduction

Numerous studies have confirmed that regular physical activity (PA) can effectively improve the physical fitness and health of children and adolescents while reducing the risk of chronic diseases such as obesity and type II diabetes (Lampard et al., 2014; Belton et al., 2014). The PA Guidelines released by the World Health Organization (WHO) in 2020 recommend that children and adolescents should engage in an average of at least 60 min of moderate-to-vigorous physical activity (MVPA) per day throughout the week (Bull et al., 2020). However, physical inactivity among children and adolescents has become a global trend, with 80% of adolescents worldwide not meeting the recommended amount of PA (Hallal et al., 2012). Only 29.9% of children and adolescents in China meet the recommended amount (Chen, 2017). Moreover, childhood and adolescence are critical periods for developing PA experiences and behavioral habits, which are closely related to their lifestyle in adulthood (Guo et al., 2017). Therefore, in-depth exploration of the influencing factors of PA in children and adolescents is an important prerequisite for accurate intervention and promotion of good behavior, and a healthy lifestyle.

The socio-ecological model indicates that an individual’s PA is influenced by multiple levels of factors, including personal, organizational, and environmental aspects (Bauman et al., 2012). Due to the characteristics of children’s physical and mental development at different stages, their PA is primarily affected by the family, school, and community environment (Welk, 1999). As the starting point of children’s growth, family environment, especially parental factors, have a crucial impact on children’s PA (Welk et al., 2003; Leung et al., 2017). Currently, numerous studies abroad indicate that parental support can have an impact on children’s PA, and it has a significant effect during the early stages of child development (before the age of 10 years) (Leung et al., 2017). However, parental support is a multidimensional factor, and research on which types of support are most closely related to children’s PA is relatively limited (Pyper et al., 2016). On one hand, although some scholars have categorized supportive behaviors, there are significant differences in the types of support due to varying classification criteria and measurement tools. For example, Lampard (Lampard et al., 2014) divided parental support into logistic support, modeling of PA and restricting access to screen-based activities. Rhodes (Rhodes et al., 2013) studied the parental role model, PA encouragement and PA participation facilitation. On the other hand, the findings from different studies are not consistent. Some studies suggest that parental PA modeling and providing convenient transportation have a positive impact on children’s PA (Wright et al., 2010; Cheng et al., 2014; Beets et al., 2010; Ploeg et al., 2013; Zhao and Settles, 2014), while other studies have found completely opposite results (Welk et al., 2003; Trost et al., 2003; Gillison et al., 2017).

The exploration of the relationship between parental factors and children’s PA in China can be traced back to the study by Cheng et al. (2009) on children in Chengdu, which started later compared to foreign research. Although in recent years, scholars in our country have strengthened research in this area (Liu et al., 2017; Hu et al., 2017; Wang and Xiao, 2018), overall development has been slow and mainly concentrated in mainland China, while studies on the impact of parental support on children’s PA in the Macau region are still relatively scarce (Zhang, 2018). The “Macau Special Administrative Region Citizen Physical Fitness Monitoring Report” highlights issues such as insufficient PA among children in Macau and the prevalence of a sedentary lifestyle with more sitting than movement (Sports Bureau Macao Special Administrative Region Government, 2016). Macau’s economy is developing rapidly, and it is the city with the highest GDP per capita and population density in China. Due to factors such as its geographical location and historical background, Macau has formed a unique culture that blends both Chinese and Portuguese elements. Therefore, exploring the relationship between parental support and children’s PA in the Macau region may result in findings that differ from those in mainland China and abroad. Additionally, due to the impact of PA recommendations, both domestic and international research has primarily focused on MVPA in children. However, existing studies have pointed out that PA of different intensities can all promote positive health benefits, with certain differences between these benefits (Laguna et al., 2013; Amagasa et al., 2018; Kwon et al., 2011). Meanwhile, the PA Guidelines for Americans scientific report also suggests that PA of different intensities should be discussed separately. However, the relationship between parental support and children’s PA at different intensities still requires further clarification. Therefore, this study aims to explore the relationship between parental support and different intensities of PA of children aged 6–10 years in Macau, and provide a theoretical basis for effective interventions of different PA intensities of children in Macau.

## 2 Methods

### 2.1 Participants

According to the relevant research design theory, 270 children in Macau were selected as research subjects. A power analysis estimated that a sample size of 100 would be sufficient. Thus this study exceeded the minimum sample size by 30 traditionally required by sociology research. According to the long-term practical experience of social survey research, the sample size of the population with an overall size of 10,000–100,000 should be between 1 and 5% (Lin and Ni, 2012). The official website of the Macau Education and Youth Affairs Bureau shows that there are a total of 24,754 children aged 6–10 in Macau (Education and Youth Bureau, Macao Special Administrative Region Government, 2018), thus the sample size meets the research requirements.

### 2.2 Procedure

Two hundred and seventy children aged 6–10 years were randomly selected in Macau. First, height and weight measurements were taken. Next, the children were guided to complete a PA survey questionnaire. Finally, parent questionnaires and informed consent forms with the school’s official stamp were distributed, to be handed over to the parents by the students. The informed consent includes the principle of voluntary completion, information confidentiality and the scientific research use of the data. After parents completed the questionnaire, students returned the questionnaire to the teachers.

A total of 270 children’s PA and parental support questionnaires were collected, with a recovery rate of 100%. After excluding non-parent responses, PA questionnaires of different intensity, missing parental support data questionnaires, and abnormal questionnaires (questionnaires with many missing items, randomly filled in and beyond the age range of this study), 223 valid questionnaires remained, with an effective recovery rate of 82.6%.

### 2.3 Variables measures

#### 2.3.1 Demographics

At the beginning of the children’s PA questionnaire, demographic information such as the child’s date of birth and gender was asked.

Height and weight measurements were conducted in the classroom by trained investigators and teachers using a height and weight measuring instrument (GMCS-IV, Jianmin, Beijing, China). During the measurement process, the children’s barefoot height was recorded to the nearest 0.1 cm, and their weight was measured to the nearest 0.1 kg. The children’s Body Mass Index (BMI) was calculated by dividing their weight by the square of their height (kg/m^2^). The BMI *z*-score was calculated based on the WHO 2007 Growth Reference (De Onis et al., 2007).

#### 2.3.2 International physical activity questionnaire-short, IPAQ-S

The International PA Questionnaire-short form was used to investigate children’s PA. According to the empirical study in Macau, this scale has good applicability, credibility, and effectiveness (Fan et al., 2014).

IPAQ-S classified PA into vigorous PA (VPA), moderate PA (MPA), and low PA (LPA), and asked participants about their frequency of participation and cumulative time per day for each of the three types of PA over the last 7 days. Due to the limited literacy and understanding ability of children, under the detailed explanation and guidance of teachers, children completed the PA survey by themselves. According to the IPAQ-S data processing rules, data cleaning and outlier elimination are carried out successively, and LPA, MPA, VPA and MVPA times are finally obtained.

#### 2.3.3 The activity support scale for multiple groups, ACTS-MG

The Parental Support Survey of children’s PA uses the parent report version of The Activity Support Scale for Multiple Groups (ACTS-MG) (Lampard et al., 2014). The ACTS-MG scale consists of 15 questions, including logistic support, PA model, community resource utilization, and restrictions on sedentary behavior. Logistical support refers to the preparation and support provided by parents for children to participate in PA, including parents to let children participate in sports teams and sports clubs, to take children to places where they can play, and to watch children participate in sports games or performances. PA model refers to parents guiding children to participate in PA by example, including family members participating in PA as a way of leisure and entertainment, parents’ attitude towards PA, participation habits, parents taking children with them when participating in PA, and encouraging children with their own example. The use of community resources is to create opportunities for children to perform PA in the community, including parents’ encouragement to exercise in the community, encourage children to walk/ride bicycles in a nearby safe and appropriate place, sign up for children to participate in community activities and provide children with school (or holidays) to participate in PA (such as summer camps). Restrictions on sedentary behavior refer to parents’ limits on the child’s static behavior time, including parents’ limits on children watching TV, using computers, mobile phones and other mobile devices. The items in the ACTS-MG are all set on a 4-point scale, where very consistent, consistent, inconsistent, and very inconsistent are assigned values of 4, 3, 2, and 1, respectively. The scores for each type of parental support are then obtained by summing these values.

ACTS-MG, as a mature scale, has been widely used in countries such as North America and Africa (Lampard et al., 2014; Chen and Dai, 2016; Yuan and Zhai, 2012). The reliability of the ACTS-MG scale was tested with a two-week interval by 115 parents of Macau children. The test–retest reliability for various types of parental support ranged between 0.534 and 0.955. The internal consistency reliability, measured by Cronbach’s alpha coefficient, was 0.928, with the dimensions of different types of parental support ranging from 0.764 to 0.942, indicating that the ACTS-MG scale has good reliability.

### 2.4 Statistical analysis

The questionnaire data were statistically analyzed using SPSS 26.0, with the significance level set at *p* < 0.05. Descriptive statistical analysis was conducted to summarize the time spent on PA of various intensities and types of parental support using means and standard deviations. Independent sample *t*-tests and one-way ANOVA were employed to analyze differences in PA times and types of parental support among children of different genders and ages. Pearson’s simple correlation was used to examine the relationship between the time spent on PA of various intensities and types of parental support among children of different genders. Finally, controlling for BMI z-score, a multilevel linear regression analysis was performed to explore the relationship between children’s PA times of various intensities and types of parental support.

## 3 Results

### 3.1 Participant characteristics

Complete and valid data were provided for 223 children out of 270 subjects, 128 boys (57.4%) and 95 girls (42.6%). The mean age of the children was (7.96 ± 1.32) years, and the mean BMI z-score of the children was 0.07. A total of 158 (70.9%) parents, who were mainly mothers, completed the ACTS-MG survey.

### 3.2 Physical activity of children

**Figure 1:**
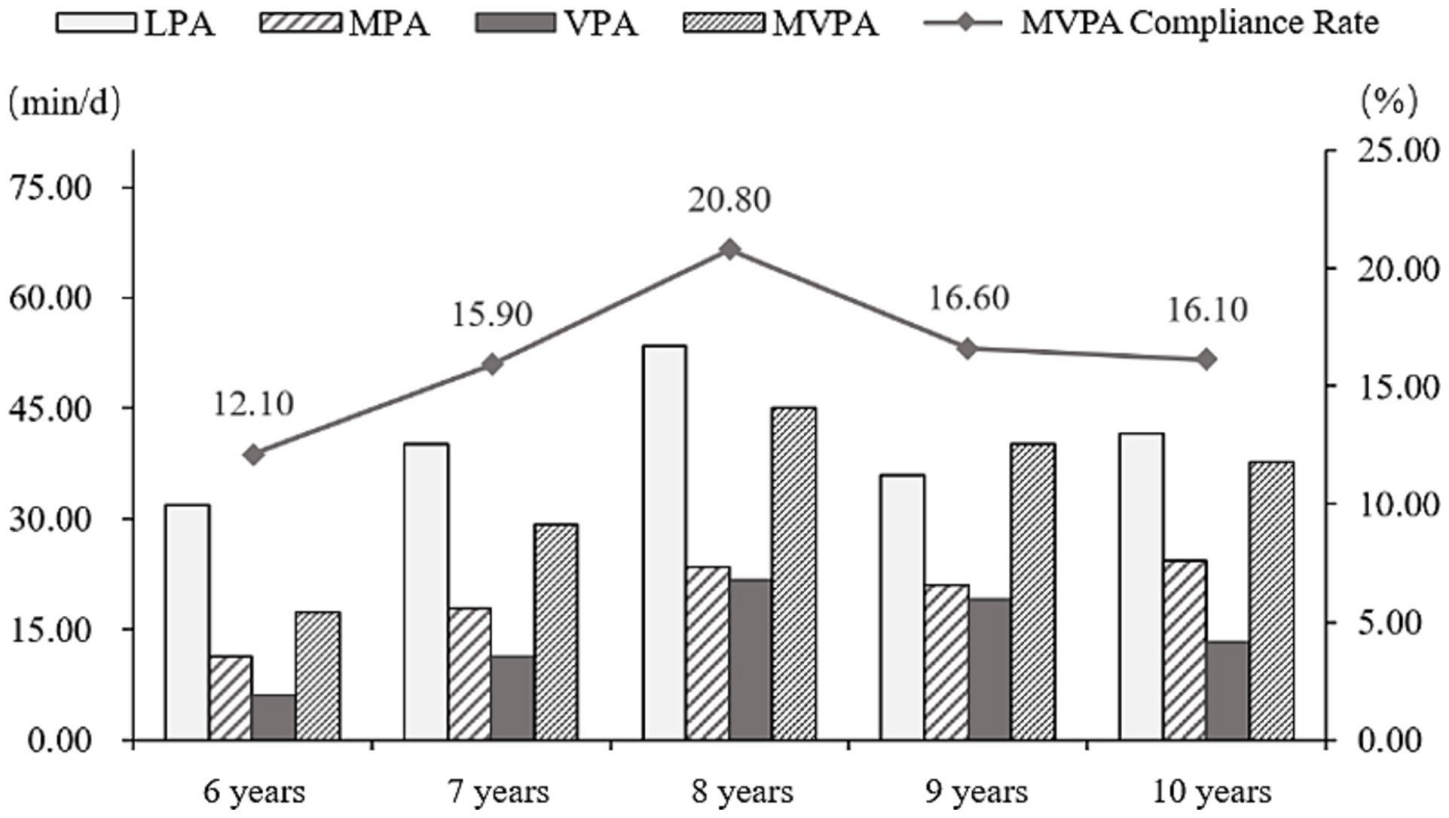
Trends in PA time of children by different age-groups MVPA compliance rate.

As shown in Table 1, the average daily LPA time for children was 41 min (41 min for boys; 41 min for girls) and 20 min for MPA (21 min; 18 min) and the VPA time was 15 min (16 min; 14 min) and MVPA time was 35 min (37 min; 32 min), there was no significant difference between boys and girls, but boys were slightly higher than girls on the whole. There was no significant difference in the PA time of children of different ages with different intensities, and it was shown in Figure 1 that the PA time of children of different intensity and the MVPA compliance rate continued to increase with age at the age of 6–8 years, but showed a downward trend at the age of 9–10 years.

**Table 1:** Children’s PA, parental support survey results (x ± s).

	*n*	BMI *z*-score	PA/min				Parental support			
			LPA	MPA	VPA	MVPA	Logistic support	PA model	Community resource utilization	Restrictions on sedentary behavior
Total	223	0.07 ± 1.65	40.96 ± 43.32	19.66 ± 30.64	14.99 ± 27.77	34.65 ± 50.91	9.03 ± 1.76	13.98 ± 2.99	11.60 ± 2.23	9.55 ± 1.63
Boys	128	0.20 ± 1.72	40.95 ± 42.42	20.69 ± 28.70	15.53 ± 24.79	36.22 ± 46.37	9.05 ± 1.78	14.00 ± 2.96	11.81 ± 2.21	9.47 ± 1.60
Girls	95	−0.11 ± 1.54	40.98 ± 44.72	18.26 ± 33.18	14.27 ± 31.46	32.53 ± 56.65	8.99 ± 1.74	13.95 ± 3.04	11.32 ± 2.22	9.66 ± 1.67
Gender		0.163	0.996	0.559	0.739	0.594	0.785	0.897	0.099	0.379
Age		0.842	0.140	0.309	0.051	0.084	0.929	0.732	0.750	0.632

### 3.3 Parental support

Parental support survey results show: The median critical value of each item of the ACTS-MG scale was 2.5 points, the sedentary behavior restriction (*M* = 3.18, SD = 0.54), logistic support (*M* = 3.01, SD = 0.58), community resource utilization (*M* = 2.90, SD = 0.56) and PA model (*M* = 2.80, SD = 0.58) in parental support type. The mean value of each item of was higher than the medium threshold, indicating that parents generally paid attention to children’s PA support, but the community resource utilization and PA models’ support levels were relatively low. In addition, Table 1 shows that there is no significant difference between different types of parental support for children of different genders and different ages, and the scores of different types of support for boys and girls are basically the same, and show a small fluctuation with the change of age.

### 3.4 Relationship between different levels of PA and parental support in children

Table 2 shows that, except that the correlation between LPA time and parental support types of boys and girls is not statistically significant, other LPA, MPA, VPA and MVPA time are statistically correlated with one or more parental support types, and the intensity of the correlation is different to some extent. They ranged from 0.141 (LPA time for PA models versus overall sample) to 0.336 (community resource utilization versus VPA time for boys). Previous studies have shown that BMI has a significant impact on children’s PA (Davison et al., 2011; Davison et al., 2003; Jago et al., 2020). Therefore, in order to further explore the relationship between the two, based on controlling BMI *z*-score, the type of parental support that has statistical significance with children’s PA time is taken as the independent variable, and the time of children’s PA with different intensity is taken as the dependent variable. The multi-layer linear regression analysis was carried out according to the stepwise forward approach.

**Table 2:** Correlation between PA time and parental support for children by different gender-groups.

	LPA			MPA			VPA			MVPA		
	Total	Boy	Girl	Total	Boy	Girl	Total	Boy	Girl	Total	Boy	Girl
Logistic support	0.136^*^	0.156	0.110	0.253^**^	0.245^**^	0.263^*^	0.250^**^	0.238^**^	0.267^**^	0.289^**^	0.279^**^	0.303^**^
PA model	0.141^*^	0.154	0.124	0.147^*^	0.106	0.193	0.264^**^	0.267^**^	0.265^**^	0.233^**^	0.208^*^	0.260^**^
Community resource utilization	0.080	0.116	0.035	0.210^*^	0.159	0.263^**^	0.286^**^	0.336^**^	0.237^*^	0.282^**^	0.278^**^	0.286^**^
Restrictions on sedentary behavior	0.094	0.093	0.097	0.011	−0.044	0.077	0.119	0.245^**^	−0.004	0.071	0.104	0.043

**Correlation is significant at the 0.01 level (two-tailed). *Correlation is significant at the 0.05 level (two-tailed).

#### 3.4.1 Relationship between LPA and parental support of child

The regression results found that parents’ logistic support and PA model had no significant effect on the overall LPA time of children, indicating that each type of parental support was not the main factor in predicting and explaining children’s LPA time (*p* > 0.05), so regression analysis and data presentation were not conducted.

#### 3.4.2 Relationship between children’s MPA, MVPA and parental support

In the MPA relationship (Table 3), overall child model 1, boy model 1 and girl model 1 show that BMI *z*-score has no significant influence on overall child MPA, boy MPA and girl MPA time, and the contribution rate is 0.4, 0.8 and 0.8%, respectively. After controlling for BMI *z*-score, logistic support, PA models and community resource utilization were included in overall child model 2, logistic support in boy model 2, and logistic support and community resource use in girl model 2. The results showed that: Logistic support was positively correlated with MPA time in children (*β* = 0.256, *p* < 0.01), MPA time in boys (*β* = 0.245, *p* < 0.05) and MPA time in girls (*β* = 0.279, *p* < 0.05), and the contribution rate increased to 5.7, 4.5 and 5.9%.

**Table 3:** Regression analysis of parental support and MPA, VPA and MVPA time for children.

Dependent Variable	Gender	Model	Variable	*R*	*R* ^2^	*R* ^2^_adj_	*B*	*SD*	*β*
MPA	Total	1		0.018	0.000	−0.004			
			BMI *z*-score				−0.339	1.251	−0.018
		2		0.256	0.065	0.057			
			BMI *z*-score				−0.665	1.215	−0.036
			logistic support				4.456	1.139	0.256^**^
			PA model				/	/	/
			community resource utilization				/	/	/
	Boys	1		0.002	0.000	−0.008			
			BMI *z*-score				0.029	1.489	0.002
		2		0.245	0.060	0.045			
			BMI *z*-score				0.001	1.449	0.001
			logistic support				3.965	1.401	0.245^**^
	Girls	1		0.053	0.003	−0.008			
			BMI z-score				−1.151	2.231	−0.053
		2		0.281	0.079	0.059			
			BMI *z*-score				−2.113	2.184	−0.098
			Logistic support				5.316	1.932	0.279^**^
			Community resource utilization				/	/	/
VPA	Total	1		0.002	0.000	−0.005			
			BMI *z*-score				−0.037	1.134	−0.002
		2		0.286	0.082	0.074			
			BMI *z*-score				0.003	1.089	0.000
			Community resource utilization				3.573	0.806	0.286^**^
			Logistic support				/	/	/
			PA model				/	/	/
	Boys	1		0.040	0.002	−0.006			
			BMI *z*-score				−0.578	1.285	−0.040
		2		0.338	0.114	0.100			
			BMI *z*-score				−0.486	1.215	−0.034
			Community resource utilization				3.756	0.944	0.335^**^
			Logistic support				/	/	/
			PA model				/	/	/
	Girls	1		0.038	0.001	−0.009			
			BMI *z*-score				0.775	2.117	0.038
		2		0.267	0.072	0.051			
			BMI *z*-score				−0.102	2.079	−0.005
			PA model				4.845	1.839	0.268^*^
			logistic support				/	/	/
MVPA	Total	1		0.012	0.000	−0.004			
			BMI *z*-score				−0.376	2.078	−0.012
		2		0.291	0.084	0.076			
			BMI *z*-score				−0.993	1.998	−0.032
			logistic support				8.428	1.873	0.291^**^
			PA model				/	/	/
			community resource utilization				/	/	/
	Boys	1		0.020	0.000	−0.008			
			BMI *z*-score				−0.548	2.405	−0.020
		2		0.280	0.078	0.064			
			BMI *z*-score				−0.601	2.318	−0.022
			logistic support				7.284	2.242	0.279^**^
			PA model				/	/	/
			community resource utilization				/	/	/
	Girls	1		0.010	0.000	−0.011			
			BMI *z*-score				−0.377	3.814	−0.010
		2		0.308	0.095	0.075			
			BMI *z*-score				−2.215	3.696	−0.060
			logistic support				10.161	3.269	0.312^**^
			PA model				/	/	/
			community resource utilization				/	/	/

**Correlation is significant at the 0.01 level (two-tailed). *Correlation is significant at the 0.05 level (two-tailed).

In relation to MVPA (Table 3), overall child model 1, boy model 1 and girl model 1 showed that BMI *z*-score had no significant influence on overall child MVPA, boy MVPA and girl MVPA time, and the contribution rates were 0.4, 0.8 and 1.1%, respectively. After controlling for BMI *z*-score, global child model 2, boy model 2, and girl model 2 all included logistic support, PA models, and community resource use, showing that: Logistic support was positively correlated with MVPA time of children (*β* = 0.291, *p* < 0.01), MVPA time of boys (*β* = 0.279, *p* < 0.05) and MVPA time of girls (*β* = 0.312, *p* < 0.05), and the contribution rate increased to 7.6, 6.4 and 7.5%.

#### 3.4.3 Relationship between child VPA and parental support

As shown in Table 3 (VPA), overall child model 1, boy model 1 and girl model 1 show that BMI *z*-score has no significant influence on overall child VPA, boy VPA and girl VPA time, and the contribution rate is 0.5, 0.6 and 0.9%, respectively. After controlling for BMI *z*-score, global child model 2 included logistic support, PA models, and community resource use, boy model 2 included logistic support, PA models, community resource use, and static behavior restriction, and girl model 2 included logistic support and PA models, and the results showed that: Community resource utilization was positively correlated with VPA time in children (*β* = 0.286, *p* < 0.01) and VPA time in boys (*β* = 0.335, *p* < 0.01), and the contribution rates were 7.4 and 10.0%, respectively. There was a significant positive correlation between PA model and VPA time of girls (*β* = 0.268, *p* < 0.05), and the contribution rate was 5.1%.

## 4 Discussion

### 4.1 Children’s physical activity

On average, children engage in 35 min of MVPA per day, with only 16.6% meeting the recommended MVPA guidelines. The rate of MVPA compliance among children in Macau is similar to the global trend of insufficient PA among children (Salin et al., 2019), and the amount of MVPA time is lower than that reported in studies conducted in mainland China and other countries (Gillison et al., 2017; Liu et al., 2017). This is closely related to the short duration of weekly PA at school and the low participation in PA outside of school among children in Macau (Sports Bureau Macao Special Administrative Region Government, 2016). Additionally, PA among Macau children continues to increase from ages 6 to 8 years, but shows a declining trend from ages 9 to 10. This result differs from foreign studies that have found a decline in PA among children at the age of 10 (Tye et al., 2020), but overall, the level of PA among these children generally shows an initial increase followed by a decrease.

This phenomenon may result from a combination of physiological, psychological, and social factors in children. Physiologically, this study found that the BMI index continues to increase with age. Existing research indicates that PA in children of normal weight is negatively correlated with changes in the BMI index (Hallal et al., 2012; Dumith et al., 2011), suggesting that the increase in BMI may inhibit children’s PA to some extent. Psychologically, based on the expectancy-value theory, children have higher expectations for PA in their early years, but these expectations gradually decline as they age, thereby affecting their activity levels (Schwarzfischer et al., 2017). Additionally, the theory of ability beliefs points out that children aged 7–12 years’ experience rapid development in social comparison skills. When children perceive their performance in PA as inferior to their peers, it can lead to self-imposed constraints on PA (Mitchell et al., 2013). Socially, the socialization of PA in early childhood is crucial and is influenced by factors such as family encouragement and recreational choices (Mitchell et al., 2013). However, interviews revealed that as Macau children grow older, parental control over their PA generally decreases, while their screen time on electronic devices significantly increases, which negatively impacts their PA to some extent.

### 4.2 Parental support

Parents generally place significant emphasis on supporting children’s PA, but the level of support for role modeling in PA and the utilization of community resources is relatively low. The findings are generally lower compared to studies abroad that used the same scale (Chen and Dai, 2016). Macau’s unique economic, cultural, and geographical environment, among other factors, have a significant impact on this phenomenon.

First of all, due to the influence of Chinese and Portuguese multi-culture, parents in Macau are relatively open to education and pay more attention to children’s PA, but the degree of attention is still low compared with foreign countries; Secondly, with the rapid economic development in Macau, parents are busy with work and often work overtime, so they have limited leisure time to exercise and accompany with their children, which fails to fully set an active example for their children (Hu et al., 2017; Xiang et al., 2003). Finally, as one of the regions with the highest population densities in the world, Macau has relatively insufficient community resources available for children’s physical activities, and children’s outdoor time is limited due to the “protection paradox” of parents (Pyper et al., 2016). In addition, the study found no gender differences in parental support. Families in Macau generally pursue “affirmative action” (Xiang et al., 2003). Women have the same social participation and status as men, and parents pay more attention to gender equality in family education and PA support.

### 4.3 The relationship between children’s physical activity and parental support

Currently, due to significant variations in the classification of parental support types and research findings (Cheng et al., 2014; Beets et al., 2010; Ploeg et al., 2013; Zhao and Settles, 2014; Gillison et al., 2017), and the relatively limited number of separate studies on parental support and children’s PA of different intensities, the relationship between them still needs further exploration and clarification. The regression analysis in this study shows that parental support does not have a significant impact on children’s LPA, while logistic support significantly affects both overall and gender-specific children’s MPA and MVPA. Additionally, the utilization of community resources has a significant impact on overall children’s VPA and boys’ VPA, and PA modeling significantly influences girls’ VPA.

The relationship between children’s LPA and parental support may be related to parents’ general support for children’s active PA. At the same time, the expectation theory points out that the expectation and demand of PA are higher in childhood, and children are more active. In addition, for Macau children, due to the narrow geographical space and nearby school, they walk to and from school with relatively few LPA and fixed time (Sports Bureau Macao Special Administrative Region Government, 2016), and the influence of parental support on children’s LPA is not significant. However, it is not that LPA in children is unimportant. Studies have pointed out that LPA for a certain period of time can effectively reduce the risk of cardiovascular disease and accidental death in children (Kwon et al., 2011). In addition, for children with an inactive lifestyle, it is not practical to directly engage in high intensity PA, and LPA is the first step to improve the level of PA of children in this group and promote health benefits. Future studies need to further explore the relevant factors affecting children’s LPA.

The significant influence factor of MPA and MVPA in children is logistical support, which may be due to relatively more PA during childhood, but compared with VPA, children are more likely to achieve MPA. At the same time, the proportion of MPA in MVPA (56%) is relatively large, so the factors affecting MPA and MVPA in children are the same. As for its potential mechanism, attachment theory points out that there is a special emotional relationship between children and parents, and parents can make children feel safe and emotional support by encouraging children to participate in sports clubs, watching children’s games and leading children to places where activities can be carried out, thus improving children’s participation interest and enthusiasm. In addition, based on the knowledge of adolescent PA promotion model (Belton et al., 2014; Welk, 1999), parents can not only directly improve children’s MPA and MVPA through logistical support, but also indirectly affect children’s self-efficacy and expected value, thus jointly improving children’s PA level.

Studies have pointed out that VPA plays an important role in preventing overweight and obesity in children (Laguna et al., 2013). This study found that there are gender differences in the influence factors of parental support on children’s VPA. On the one hand, because VPA is difficult to achieve and can cause shortness of breath and rapid heartbeat, children are prone to fear of performing vigorous activities (Geng and Liang 2008). On the other hand, role theory suggests that gender role stereotypes can influence individual goals, expectations, and social experiences, leading boys and girls to participate in gender-appropriate activities. For example, boys tend to play basketball, soccer, and other sports with high confrontation and intensity, while girls tend to play physical sports with low confrontation and intensity. Therefore, there are significant differences between boys and girls in their ability perception and expected value of VPA (Hu et al., 2017; Mitchell et al., 2013). In addition, social learning theory points out that observational learning and imitative behavior are dominant in childhood, and children will imitate the behaviors of important people such as parents and peers when participating in physical activities (Mitchell et al., 2013). Therefore, combining role theory and social learning theory, parents can provide community resource utilization support in the form of participating in sports clubs or summer camps in the community, so that boys can get more free space and be easily influenced by peers, which is more conducive to improving the expected value and level of VPA for boys.

In general, parental support in Macau can have an impact on children’s PA (except LPA), and the impact on different intensity of PA is different. Since the return of Macau to China, great changes have taken place in many aspects of society, and the family structure tends to be a small nuclear family model. Parents in nuclear families pay more attention to sports participation and parent–child activities, which has a positive impact on children’s PA (Xiang et al., 2003). In addition, although the static behavior restriction score of parental support in Macau is high, the study found that it does not indirectly translate into PA promotion, and the direct support of parents for PA is more conducive to promoting the improvement of children’s PA level.

The contribution of parental support to MPA, VPA and MVPA in boys is between 5 and 10%. The results of this study are basically consistent with the findings of mainland children (Wang and Xiao, 2018), but compared with foreign studies (10–40%), parental support in Macau has a lower role in explaining and predicting children’s PA (Trost et al., 2003; Yuan and Zhai, 2012; Wu et al., 2009). Due to the profound influence of China’s traditional culture and educational concepts, Macau parents have higher expectations for cultural knowledge education than foreign countries, and it is easy to make children have wrong sports cognition concepts, resulting in Macau parents’ support for children’s PA interpretation degree is relatively limited. In addition, social ecology model (Bauman et al., 2012) and adolescent PA promotion model (Belton et al., 2014) both point out that PA of children and adolescents is influenced by multiple factors. Besides parental support, factors such as individual characteristics, family environment, school environment, community environment and policies also have an important impact on children’s PA. Future studies should comprehensively analyze the contribution of parental support to children’s PA based on these factors.

## 5 Limitations and implications

This study has several limitations that should be acknowledged. First, its cross-sectional design prevents causal inference between parental support and children’s PA. Second, self-reported measures may involve recall or social desirability bias. Third, while the sample size exceeded the minimum requirements for social research, the study was limited to children aged 6–10 years in Macau, restricting the generalizability of the findings to other age groups or regions with different socio-cultural environments.

Despite these limitations, the study provides useful insights. It shows that different types of parental support relate differently to PA intensities, with logistical support especially important for MVPA. Interventions should target specific forms of support, tailored by age, gender, and context. Policymakers and practitioners should consider family-centered approaches and work to improve community and school environments to encourage children’s physical activity at all levels.

## 6 Conclusion

Parents in Macau generally pay attention to the PA support of children, but the PA level of children is relatively low and shows a trend of increasing first and then decreasing with the increase of age. It is recommended that Macau parents further raise the level of support, especially at the age of 8 years, to attract sufficient attention to lay the foundation for PA promotion in adolescence and adulthood. There are some differences in the influence of parental support types on PA intensity of children of different genders in Macau, and the relationship between parents’ logistic support and children’s PA is closer. When family intervention is recommended, it is necessary to develop targeted plans and measures to stimulate the participation interest and enthusiasm of boys and girls in a more effective way to meet the exercise needs of different intensities of PA.

## Data Availability

The original contributions presented in the study are included in the article/Supplementary material, further inquiries can be directed to the corresponding author/s.

## References

[ref1] Amagasa S. Machida M. Fukushima N. Kikuchi H. Takamiya T. Odagiri Y.. (2018). Is objectively measured light-intensity physical activity associated with health outcomes after adjustment for moderate-to-vigorous physical activity in adults? A systematic review. Int. J. Behav. Nutr. Phys. Act. 15:65. doi: 10.1186/s12966-018-0695-z, PMID: 29986718 PMC6038338

[ref2] Bauman A. E. Reis R. S. Sallis J. F. Wells J. C. Loos R. J. Martin B. W.. (2012). Correlates of physical activity: why are some people physically active and others not? Lancet 380, 258–271. doi: 10.1016/S0140-6736(12)60735-1, PMID: 22818938

[ref3] Beets M. W. Cardinal B. J. Alderman B. L. (2010). Parental social support and the physical activity-related behaviors of youth: a review. Health Educ. Behav. 37, 621–644. doi: 10.1177/1090198110363884, PMID: 20729347

[ref4] Belton S. Brien W. O. Meegan S. O’ Brien W. Woods C. Issartel J. (2014). Youth-physical activity towards health: evidence and background to the development of the Y-path physical activity intervention for adolescents. BMC Public Health 14:122. doi: 10.1186/1471-2458-14-122, PMID: 24499449 PMC3922546

[ref5] Bull F. C. Al-Ansari S. S. Biddle S. Borodulin K. Buman M. P. Cardon G.. (2020). World Health Organization 2020 guidelines on physical activity and sedentary behaviour. Br. J. Sports Med. 54, 1451–1462. doi: 10.1136/bjsports-2020-102955 33239350 PMC7719906

[ref6] Chen P. (2017). Physical activity, physical fitness, and body mass index in the Chinese child and adolescent populations: an update from the 2016 physical activity and fitness in China—the youth study. J. Sport Health Sci. 6, 381–383. doi: 10.1016/j.jshs.2017.09.011, PMID: 30356661 PMC6189246

[ref7] Chen H. Dai J. (2016). Does gender moderate the direct and indirect relationships between different sources of social support and adolescents' physical activity? J. Phys. Act. Health 13, 874–881. doi: 10.1123/jpah.2015-0547, PMID: 27144409

[ref8] Cheng L. A. Mendonça G. Farias Júnior J. C. (2014). Physical activity in adolescents: analysis of the social influence of parents and friends. J. Pediatr. 90, 35–41. doi: 10.1016/j.jped.2013.05.006, PMID: 24156835

[ref9] Cheng G.-P. Zeng G. Liu J. Feng M. Guo H.-X. (2009). Influence of family environment on physical activity of school-age children. Chin. J. Sch. Health, 30, 903–904. doi: 10.16835/j.cnki.1000-9817.20091118

[ref10] Davison K. K. Cutting T. M. Birch L. L. (2003). Parents' activity-related parenting practices predict girls' physical activity. Med. Sci. Sports Exerc. 35:1589. doi: 10.1249/01.MSS.0000084524.19408.0C, PMID: 12972881 PMC2530913

[ref11] Davison K. K. Li K. Baskin M. L. Cox T. Affuso O. (2011). Measuring parental support for children's physical activity in white and African American parents: the activity support scale for multiple groups (Acts-mg). Prev. Med. 52, 39–43. doi: 10.1016/j.ypmed.2010.11.008, PMID: 21111755 PMC3022380

[ref12] De Onis M. Onyango A. W. Borghi E. Siyam A. Nishida C. Siekmann J. (2007). Development of a who growth reference for school-aged children and adolescents. Bull. World Health Organ. 85, 660–667. doi: 10.2471/BLT.07.043497 18026621 PMC2636412

[ref14] Dumith S. C. Gigante D. P. Domingues M. R. Kohl H. W. (2011). Physical activity change during adolescence: a systematic review and a pooled analysis. Int. J. Epidemiol. 40, 685–698. doi: 10.1093/ije/dyq272, PMID: 21245072

[ref15] Education and Youth Bureau, Macao Special Administrative Region Government. (2018). Educational statistics[Eb/Ol]. Available online at: http://portal.dsej.gov.mo/webdsejspace/internet/category/teachorg/Inter_main_page.jsp?id=8525# (Accessed January 1, 2025).

[ref16] Fan M. Lu J. He P. (2014). Calculation method of physical activity level in international physical activity questionnaire. Chin. J. Epidemiol. 35, 961–964. doi: 10.3760/cma.j.issn.0254-6450.2014.08.019 25376692

[ref17] Geng P.-X. Liang G.-L. (2008). Introduction to human movement development. Beijing: People’s Education Press, 345–367.

[ref18] Gillison F. B. Standage M. Cumming S. P. Zakrzewski-Fruer J. Rouse P. C. Katzmarzyk P. T. (2017). Does parental support moderate the effect of children's motivation and self-efficacy on physical activity and sedentary behaviour? Psychol. Sport Exerc. 32, 153–161. doi: 10.1016/j.psychsport.2017.07.004

[ref19] Guo Q. Wang X. Jiang J. (2017). Study on physical activity and sedentary behavior patterns of Chinese children and adolescents. Sports Sci., 37,17–29. doi: 10.16469/j.css.201707003

[ref20] Hallal P. C. Andersen L. B. Bull F. C. Guthold R. Haskell W. Ekelund U.. (2012). Global physical activity levels: surveillance progress, pitfalls, and prospects. Lancet 380, 247–257. doi: 10.1016/S0140-6736(12)60646-1, PMID: 22818937

[ref21] Hu Y.-Y. Tang Y. Zhang J. Liu Y. (2017). Study on the influence of parental factors on moderate to vigorous physical activity of adolescents. China Sport Sci. Technol. 53, 14–21.

[ref22] Jago R. Salway R. Emm-Collison L. Sebire S. J. Thompson J. L. Lawlor D. A. (2020). Association of Bmi category with change in children's physical activity between ages 6 and 11 years: a longitudinal study. Int. J. Obes. 44, 104–113. doi: 10.1038/s41366-019-0459-0, PMID: 31712707 PMC6923172

[ref23] Kwon S. Janz K. F. Burns T. L. Levy S. M. (2011). Association between light-intensity physical activity and adiposity in childhood. Pediatr. Exerc. Sci. 23, 218–229. doi: 10.1123/pes.23.2.218, PMID: 21633134 PMC3137912

[ref24] Laguna M. Ruiz J. R. Lara M. T. Aznar S. (2013). Recommended levels of physical activity to avoid adiposity in Spanish children. Pediatr. Obes. 8, 62–69. doi: 10.1111/j.2047-6310.2012.00086.x, PMID: 22961693

[ref25] Lampard A. M. Nishi A. Baskin M. L. Carson T. L. Davison K. K. (2014). The activity support scale for multiple groups (Acts-mg): child-reported physical activity parenting in African American and non-Hispanic white families. Behav. Med. 42, 112–119. doi: 10.1080/08964289.2014.979757, PMID: 25350515 PMC4412752

[ref26] Leung K.-M. Chung P.-K. Kim S. (2017). Parental support of children's physical activity in Hong Kong. Eur. Phys. Educ. Rev. 23, 141–156. doi: 10.1177/1356336X16645235

[ref27] Lin J. Ni A. (2012). Outline of social investigation and research methods. Jinan: Shandong People’s Publishing House.

[ref28] Liu Y. Zhang Y. Chen S. Zhang J. Guo Z. Chen P. (2017). Associations between parental support for physical activity and moderate-to-vigorous physical activity among Chinese school children: a cross-sectional study. J. Sport Health Sci. 6, 410–415. doi: 10.1016/j.jshs.2017.09.008, PMID: 30356620 PMC6189258

[ref29] Mitchell J. A. Pate R. R. España-Romero V. O'Neill J. R. Dowda M. Nader P. R. (2013). Moderate-to-vigorous physical activity is associated with decreases in body mass index from ages 9 to 15 years. Obesity 21, E280–E286. doi: 10.1002/oby.20118, PMID: 23592682

[ref30] Ploeg K. A. V. Kuhle S. Maximova K. McGavock J. Wu B. Veugelers P. J. (2013). The importance of parental beliefs and support for pedometer-measured physical activity on school days and weekend days among Canadian children. BMC Public Health. 13, 1–7. doi: 10.1186/1471-2458-13-1132 24308428 PMC4234294

[ref31] Pyper E. Harrington D. Manson H. (2016). The impact of different types of parental support behaviours on child physical activity, healthy eating, and screen time: a cross-sectional study. BMC Public Health 16:568. doi: 10.1186/s12889-016-3245-0, PMID: 27554089 PMC4995744

[ref32] Rhodes R. E. Berry T. Craig C. L. Faulkner G. Latimer-Cheung A. Spence J. C.. (2013). Understanding parental support of child physical activity behavior. Am. J. Health Behav. 37, 469–477. doi: 10.5993/AJHB.37.4.5, PMID: 23985228

[ref34] Salin K. Huhtiniemi M. Watt A. Hakonen H. Jaakkola T. (2019). Differences in the physical activity, sedentary time, and Bmi of Finnish grade 5 students. J. Phys. Act. Health 16, 765–771. doi: 10.1123/jpah.2018-0622, PMID: 31310997

[ref35] Schwarzfischer P. Weber M. Gruszfeld D. Socha P. Luque V. Escribano J.. (2017). Bmi and recommended levels of physical activity in school children. BMC Public Health 17:595. doi: 10.1186/s12889-017-4492-4, PMID: 28645324 PMC5482946

[ref36] Sports Bureau Macao Special Administrative Region Government (2016). Health monitoring report of citizens in Macao Special Administrative Region. Large Form Advertising Co., 72–145.

[ref37] Trost S. G. Sallis J. F. Pate R. R. Freedson P. S. Taylor W. C. Dowda M. (2003). Evaluating a model of parental influence on youth physical activity. Am. J. Prev. Med. 25, 277–282. doi: 10.1016/S0749-3797(03)00217-4, PMID: 14580627

[ref38] Tye L. S. Scott T. Haszard J. J. Peddie M. C. (2020). Physical activity, sedentary behaviour and sleep, and their association with Bmi in a sample of adolescent females in New Zealand. Int. J. Environ. Res. Public Health. 17, 6346. doi: 10.3390/ijerph17176346 32878296 PMC7503577

[ref39] Wang L. Xiao Y. (2018). The influence of parental factors on children’s leisure time physical activity: from the perspective of gender difference. J. Shanghai Univ. Sport. 42, 79–86. doi: 10.16099/j.sus.2018.01.012

[ref40] Welk G. J. (1999). The youth physical activity promotion model: a conceptual bridge between theory and practice. Quest 51, 5–23. doi: 10.1080/00336297.1999.10484297

[ref41] Welk G. J. Wood K. Morss G. (2003). Parental influences on physical activity in children: an exploration of potential mechanisms. Pediatr. Exerc. Sci. 15, 19–33. doi: 10.1123/pes.15.1.19

[ref42] Wright M. S. Wilson D. K. Griffin S. Evans A. (2010). A qualitative study of parental modeling and social support for physical activity in underserved adolescents. Health Educ. Res. 25, 224–232. doi: 10.1093/her/cyn043, PMID: 18703530 PMC2900883

[ref43] Wu S. Lv S. Zhai Q. Yang Z.-H. Shang Z.-N. Zhou J.-G. (2009). Investigation and analysis of family sports status in Macao. J. Shanghai Sport Univ. 33, 11–17. doi: 10.16099/j.cnki.jsus.2009.06.004

[ref44] Xiang P. Mcbride R. Guan J. Solmon M. (2003). Children’s motivation in elementary physical education: an expectancy-value model of achievement choice. Res. Q. Exerc. Sport. 74, 25–35. doi: 10.1080/02701367.2003.10609061 12659473

[ref45] Yuan J. Zhai Q. (2012). Comparative analysis of students' participation level in physical activity, vol. 3. Macao: Macao Polytechnic Institute.

[ref46] Zhang Q. (2018). A comparative study on physical activity of college students and senior high school students in Macao. J. Chengdu Univ. Phys. Educ. 44, 98–102. doi: 10.15942/j.jcsu.2018.04.016

[ref47] Zhao J. Settles B. H. (2014). Environmental correlates of children's physical activity and obesity. Am. J. Health Behav. 38, 124–133. doi: 10.5993/AJHB.38.1.13, PMID: 24034687

