# Supplementary material for: Association of different intensities of physical activity in children with parental support
Source: Front Psychol. 2025 Jul 21;16:1600667. doi: 10.3389/fpsyg.2025.1600667 (PMC12319029; doi:10.3389/fpsyg.2025.1600667)
Supplement: Supplementary file 1 [file Data_Sheet_1.zip › Data & Consent Form/Consent Form.docx]

**Parental or Guardian Informed Consent Form**

Dear Parent or Guardian：

Hello, I am Dr. Li Kai from Shanghai University of Sport. I aim to understand your child's level of physical activity and the level of parental support. Therefore, we need both you and your child to complete a questionnaire separately. The questionnaire process will not cause any harm to your child.

Participation in this study is entirely voluntary. Both the child and the parent/guardian can decide whether to participate. Students who do not participate in the study will continue their classes as usual without any impact. The data collected in this study will only be used for overall statistical analysis, and individual test results will be kept strictly confidential.

Please fill out the Parental or Guardian Informed Consent Form and have your child return it to the teacher at school.

We sincerely appreciate your cooperation and support!

School Name：

Student Name：

Student Class：

- Please check“✔”the option that applies to you:
- I agree for my child to participate in the study.
- I do not agree for my child to participate in the study.

Parent/Guardian Signature:
